# Gray matter morphological anomalies in the cerebellar vermis in first-episode schizophrenia patients with cognitive deficits

**DOI:** 10.1186/s12888-017-1543-4

**Published:** 2017-11-22

**Authors:** Jingjuan Wang, Li Zhou, Chunlei Cui, Zhening Liu, Jie Lu

**Affiliations:** 1 Department of Nuclear Medicine, Xuanwu Hospital Capital Medical University, 45 Changchun Street, Beijing, 100053 China; 2 Institute of Mental Health, Second Xiangya Hospital of Central South University, Changsha, Hunan China; 3 State Key Laboratory of Medical Genetics, Central South University, Changsha, Hunan China

**Keywords:** First-episode schizophrenia, Gray matter density, Voxel-based morphometry, Cerebellar vermis, Cognitive deficits

## Abstract

**Background:**

Cognitive deficits are a core feature of early schizophrenia. However, the pathological foundations underlying cognitive deficits are still unknown. The present study examined the association between gray matter density and cognitive deficits in first-episode schizophrenia.

**Method:**

Structural magnetic resonance imaging of the brain was performed in 34 first-episode schizophrenia patients and 21 healthy controls. Patients were divided into two subgroups according to working memory task performance. The three groups were well matched for age, gender, and education, and the two patient groups were also further matched for diagnosis, duration of illness, and antipsychotic treatment. Voxel-based morphometric analysis was performed to estimate changes in gray matter density in first-episode schizophrenia patients with cognitive deficits. The relationships between gray matter density and clinical outcomes were explored.

**Results:**

Patients with cognitive deficits were found to have reduced gray matter density in the vermis and tonsil of cerebellum compared with patients without cognitive deficits and healthy controls, decreased gray matter density in left supplementary motor area, bilateral precentral gyrus compared with patients without cognitive deficits. Classifier results showed GMD in cerebellar vermis tonsil cluster could differentiate SZ-CD from controls, left supplementary motor area cluster could differentiate SZ-CD from SZ-NCD. Gray matter density values of the cerebellar vermis cluster in patients groups were positively correlated with cognitive severity.

**Conclusions:**

Decreased gray matter density in the vermis and tonsil of cerebellum may underlie early psychosis and serve as a candidate biomarker for schizophrenia with cognitive deficits.

## Background

Cognitive deficits are one of the main symptoms in early schizophrenia [1] and are especially marked in sustained attention, learning, processing speed, language, motor skills, and working memory. Based on the limited impact of conventional antipsychotics on cognitive symptoms [2], there has been an interest in developing pharmacological treatments to target cognitive deficits. In addition, studies have shown that differences across abnormal types might be generated by different mechanisms [3]. Investigating the neural substrates of cognitive deficits is a clinically important endeavor that would provide insight into understanding cognitive deficits and new therapeutic targets.

Across multiple studies, patients who have relatively intact cognition comprise 20–25% of schizophrenia patients [4]. However, the wide heterogeneity of schizophrenia patients could partially confuse the final conclusions of such studies. Some related studies have focused on cognitive deficits in schizophrenia. Based on a simplified comparison of brain regions, significantly smaller white matter volumes and larger lateral ventricle volumes have been found in schizophrenia patients with cognitive deficits relative to healthy subjects, but not in patients without cognitive deficits [5]. However, whole-brain voxel-based morphometry (VBM) failed to reveal a significant difference in gray and white matter volume between two subgroups of schizophrenia patients with 23 years of illness who were divided by cognitive ability [6]. Furthermore no significant difference was found in white matter integrity between schizophrenia patients with and without cognitive deficits based on diffusion tensor imaging [7].

Although prior studies have considered symptom diversity as an influential factor, there has been inconsistency among findings. To obtain more reliable results, we made improvements in the study design from two aspects. First, the subgroup derivation rules were updated. Based on task accuracy, patients were divided into two subgroups: 1) schizophrenia patients with cognitive deficits (SZ-CD); 2) schizophrenia patients with no cognitive deficits (SZ-NCD). In addition, we also validated the results using other clinical assessments. Second, we recruited first-episode schizophrenia patients to minimize the confounding factor of illness course and antipsychotic medication use.

In the present study, we modified the subgroup derivation rules to recruit eighteen first-episode SZ-CD patients, sixteen first-episode SZ-NCD patients, and twenty-one healthy controls (HCs). Gray matter density (GMD) had been reported to be more consistent and sensitive in the schizophrenia patients [8] and characterized as brain structure biomarkers for the psychosis biotypes [9]. We focused on characterizing changes in GMD in first-episode schizophrenia patients with and without cognitive deficits.

## Methods

### Subjects

We recruited fifty-eight right-handed participants. Among them, thirty-seven first-episode patients determined by the Structured Clinical Interview for Diagnostic and Statistical Manual for Mental Disorders Fourth Edition (DSM-IV) Axis I Disorders, Patient Edition (SCID-I/P), were recruited from the Department of Psychiatry, Second Xiangya Hospital of Central South University, Changsha, China. We required all patients to have at least 9 years of education, to have been diagnosed with schizophrenia within the past 18 months, and to have no history of chronic neurological disease, substance abuse, electroconvulsive therapy, severe medical disorder or antidepressant. In the month before functional Magnetic Resonance Imaging (fMRI), the positive and negative symptoms of each patient were independently assessed using the Scale for Assessment of Positive Symptoms (SAPS) and the Scale for the Assessment of Negative Symptoms (SANS). Additionally, we also evaluated the cognitive function of all participants by using a parametric n-back task during the fMRI scan.

Twenty-one right-handed HCs matched for age, sex, and years of education were recruited from the city of Changsha and were without past or present psychiatric, neurological, or other neurological disorders, as determined by an abbreviated version of the Comprehensive Assessment of Symptoms and History [7]. Informed consent was fully explained to each participant, who then each gave written informed consent. Demographics and clinical characteristics of all participants are shown in Table 1.

**Table 1:** Demographic and clinical characteristics of three groups

Demographics	HCs	SZ-NCD	SZ-CD	P
	10 M:11 F	7 M:11 F	11 M:5 F	0.205
	Mean ± SD	Mean ± SD	Mean ± SD	
Age(years)	22.38 ± 3.94	24.5 ± 6.70	22.63 ± 6.71	0.71
Education(years)	13.33 ± 1.83	12.39 ± 2.06	12.16 ± 2.64	0.78
WAIS-Digital	–	71.53 ± 1.61	58.69 ± 12.32	0.02*
WAIS-Information	–	18.92 ± 5.13	15.1 ± 4.26	0.03*
Duration of illness (months)	–	8.25 ± 5.69	8.27 ± 4.31	0.83
CPZ equivalents	–	238.89 ± 104.16	375.12 ± 328.43	0.14
SAPS total	–	18.06 ± 8.62	16.23 ± 8.46	0.11
SANS total	–	21.11 ± 23.74	30.26 ± 2.54	0.28
Accuracy (%)^a^				
2back	84.60 ± 8.28 75~94	73.61 ± 7.62 64~96	54.78 ± 9.26 40~72	-
0back	97.31 ± 1.85 92~100	95.89 ± 5.52 78~100	77.36 ± 8.35 64~98	
Reaction time (ms)^b^				
2back	625.60 ± 120.14 427.72~911.41	692.35 ± 119.49 549.6~944.74	729.35 ± 205.07 581.02~1056.8	-
0back	482.29 ± 50.22 383.46~585.06	529.27 ± 76.18 429.37~719.65	553.11 ± 114.04 466.47~857.02	

*SD* standard deviation, *WAIS-Digital* the Digit Symbol Substitution Test of the Wechsler Adult Intelligence Scale, *WAIS-Information* the information subscale of the Wechsler Adult Intelligence Scale, *CPZ* chlorpromazine, *SAPS* Scale for Assessment of Positive Symptoms, *SANS*, Scale for the Assessment of Negative Symptoms

*p<0.05 significant difference was found

^a^The raw cognitive scores were calculated as the average of the Target and Non-target accuracies in N back tasks

^b^Reaction time was calculated as the average of the Target and Non-target reaction time in N back tasks

Part of patients and healthy controls have been choose to investigate disrupted effective connectivity in schizophrenia patients [10],while Zhou et al. [11] have choose other part of patients to provide evidence for inefficient default mode network (DMN) suppression in schizophrenia with cognitive deficits. This study was approved by the Ethics Committee of the Second Xiangya Hospital, Central South University, Hunan, China.

### N-back task

A letter n-back task was performed on NordicNeurolab’s fMRI hardware system as previously described [10]. Briefly, the task included two alternating conditions: the ‘0-back’ condition and the ‘2-back’ condition. In the 0-back condition, subjects pressed the right button if the letter x appeared; otherwise, the left button was pressed. In the 2-back condition, subjects pressed the right button when the letter was identical to the one presented two trials previously; otherwise, the left button was pressed. The scan session comprised four 2-back and four 0-back blocks for 8 min and 20 s. Each block was preceded by a text instruction shown for 2 s; participants were then shown 20 trials, of which 7 trials were the target trials. Each trial was presented for 500 ms, followed by an inter-letter interval of 1500 ms. Before the real task, all participants needed practice to reach the required accuracy of correct responses 80%.

### Statistical analysis

Three patients with significant head motion (maximum motion larger than 2° or 2 mm) or other artifacts were discarded. The remaining patients were placed into either the SZ-CD group or the SZ-NCD group on the basis of their accuracy in the 2-back task. The raw cognitive scores were calculated as the average of the Target and Non-target accuracies in the 2-back task. Then, each raw cognitive score was normally distributed with a mean of 0 and a standard deviation of 1. Patients were placed in the SZ-CD group if their normalized cognitive score was more than 1 SD below the normative mean [7]. The approach resulted in eighteen patients assigned to the SZ-CD group and sixteen patients to the SZ-NCD group.

Furthermore, the Digital Symbol Substitution Test (DSST) and the Information Subscale of Wechsler Adult Intelligence Scale Chinese Revised (WAIS-CR) [10, 12] (Table 1) were used to measure two other important cognitive functions, information processing speed and verbal comprehension, respectively. There were significant differences between the SZ-CD and SZ-NCD groups in information processing speed and verbal comprehension as assessed by a two-sample t-test using IBM SPSS statistics (version 20.0), providing further support for our criteria.

### Image acquisition

All high-resolution T1-weighted images were acquired in the Department of Psychiatry, Second Xiangya Hospital of Central South University, Changsha, China, using a T1-weighted 3D turbo field echo on a Philips Gyroscan Achieva 3.0 Tesla MRI scanner with the following parameters: repetition time = 7.5 ms, echo time = 3.7 ms, flip angle = 8°, field of view = 240 × 240 mm^2^, acquisition matrix =256 × 200, voxel resolution 1.0 × 1.0 × 1.0 mm^3^,slice thickness = 1 mm, gap = 0, and number of slices = 180. Functional MRI images were collected in the axial direction, using a gradient-echo echo planar imaging (EPI) sequence: repetition time (TR) = 2000 ms, echo time (TE) = 30 ms, matrix = 64 × 64, slices = 36, slice thickness = 4 mm, gap =0 mm, flip angle = 90°, FOV 24 × 24 cm, 250 time points. The scan lasted for 8min20s.Foam pads and earplugs were used to minimize head motion and scanner noise. All scans were inspected for quality control.

### Image processing

Structural images analysis was performed using the VBM8 toolbox version 1.19, an extension of the Statistical Parametric Mapping 8 (SPM8) software package. The main procedure included the following steps: 1) checking for scanner artifacts and gross anatomical abnormalities for each subject; 2) setting the image origin to the anterior commissure; 3) segmenting the images into gray matter and white matter images; 4) using the DARTEL toolbox on SPM8 to produce a high-dimensional normalization protocol; and 5) smoothing the unmodulated gray matter images with a Gaussian kernel of 8-mm full-width at half-maximum (FWHM).

Voxel-wise differences in the GMD in the intracerebral cortex were assessed using voxel-wise one way analysis of variance (ANOVA) across the three subject groups after controlling for age and gender(voxel wise *p* < 0.05 and cluster size >3554voxels). Multiple comparisons were corrected using Monte Carlo simulations in the Resting-State fMRI Data Analysis Toolkit (REST, http://rest.restfmri.net). Minimum cluster size was determined by AlphaSim. Restricted to the above voxels identified by ANOVA, between-group differences in gray matter densities were tested using post-hoc tests. The same threshold adjustment method was used, SZ-CD and HCs (voxel size *p* < 0.05, cluster size > 3152voxels), SZ-NCD and HCs (voxel size *p* < 0.05, cluster size > 3369voxels), SZ-CD and SZ-NCD (voxel size p < 0.05, cluster size >3380 voxels), which were equivalent to an AlphaSim correction threshold of *p* < 0.05. For visual observation, the significant voxels were overlaid onto a high-definition T1-weighted brain template to show group differences.

The average morphological anomalies in each significant cluster from both SZ patient groups and HCs were extracted. GMD comparisons among the three groups and between each pair of groups were also assessed by ANOVA using IBM SPSS statistics (version 20.0). Receive operation curve (ROC) could illustrate the diagnostic ability of a binary classifier system when different discrimination threshold was set, which was usually used to differentiate the patients from the healthy controls or between subgroups. Furthermore, we plotted ROC to explore whether the GMD values in each significant cluster could differentiate the patients from the healthy controls or between subgroups.

Voxel–wise Pearson correlation coefficients were calculated between the GMD values of significant clusters and N-back performance scores, duration of illness and medication doses to examine potential associations between morphological deficits and clinical symptoms in the combined SZ-CD and SZ-NCD groups after controlling age and sex. Correlation analyses were performed in the Resting-State fMRI Data Analysis Toolkit with a corrected *p* < 0.05 (*p* < 0.05, cluster size >4484mm^3^).

## Results

### Demographic and clinical characteristics

Among the three participant groups, no significant differences in age, years of education, or gender were found by ANOVA or chi-square tests (Table 1). There were also no significant differences in the duration of illness, medication dosage, SANS, or SAPS between the SZ-CD and SZ-NCD patient groups. By contrast, a two-sample t-test showed that both information processing speed and verbal comprehension differed significantly between the SZ-CD and SZ-NCD groups.

### Brain structural differences

**Fig. 1:**
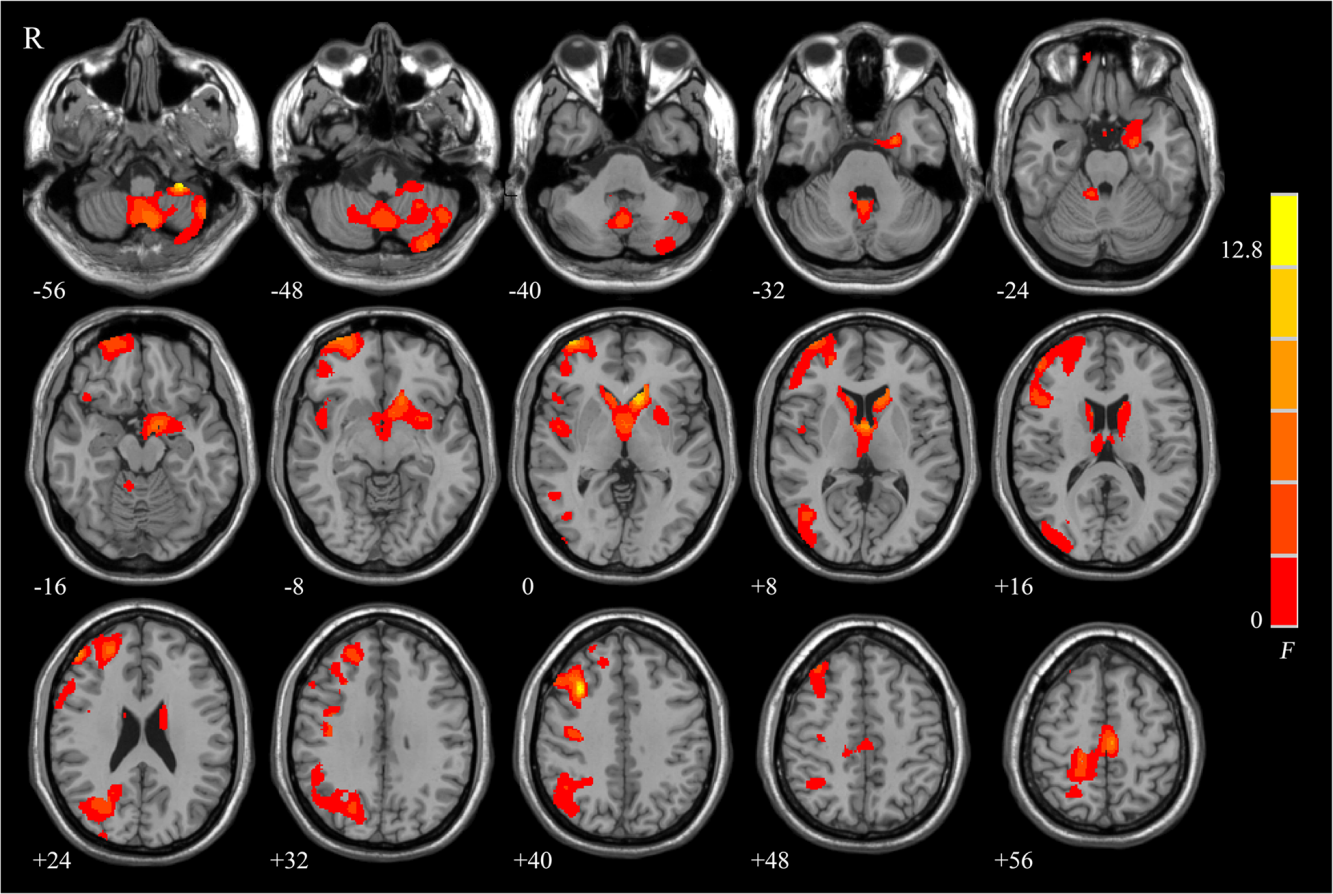
Significant group differences among the three groups are shown. These significant regions are shown as color-coded statistical F-values superimposed on 3D slices of the human brain

**Fig. 2:**
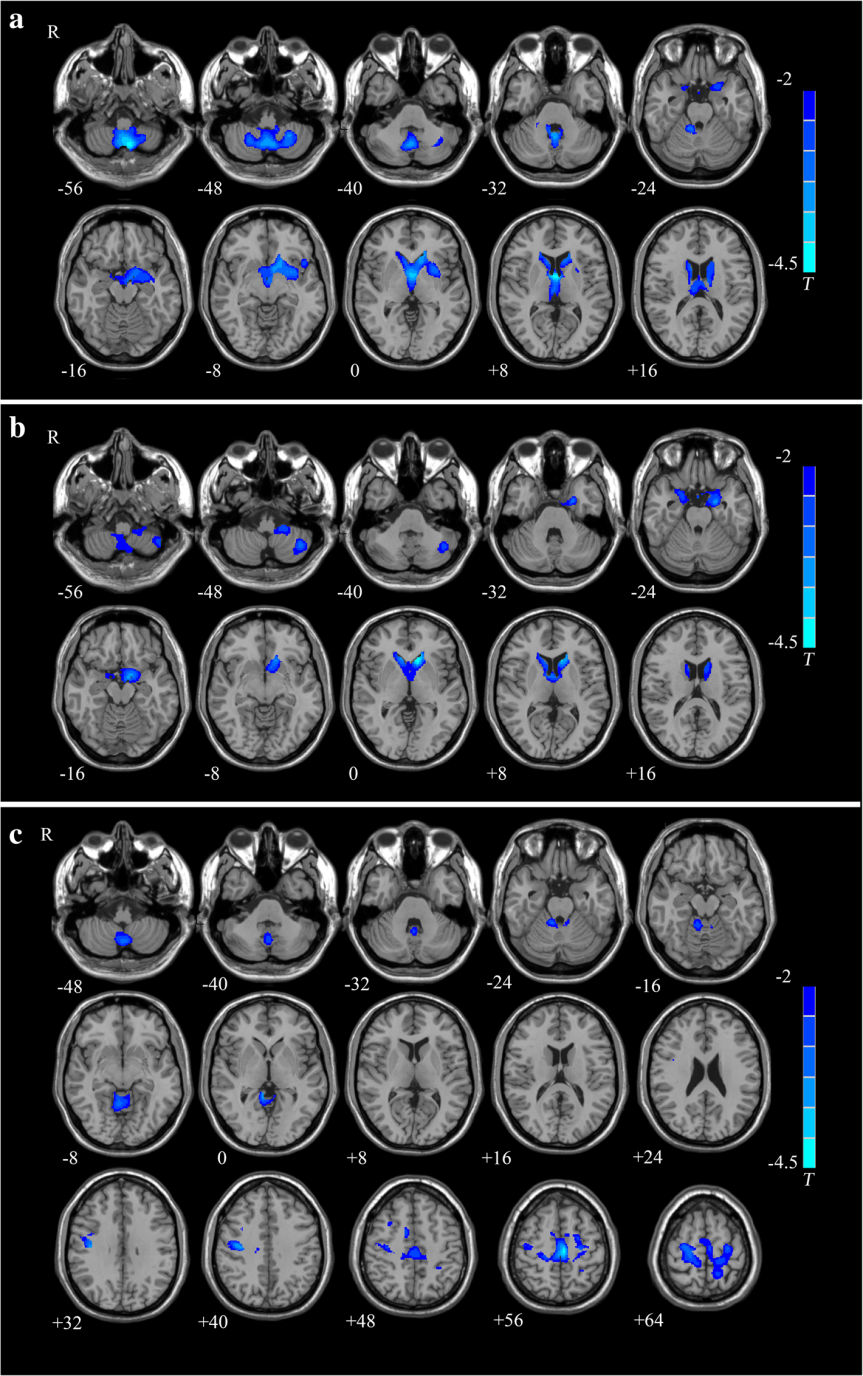
Significant group differences of two patients groups compared to healthy controls were identified. **a** Patients with cognitive deficits vs healthy controls, *P* < 0.05, corrected for multiple comparisons, cluster size >3152 voxels and. **b** Patients without cognitive deficits vs healthy controls. *P* < 0.05, corrected for multiple comparisons, cluster size >3369 voxels. **c** Patients with cognitive deficits vs Patients without cognitive deficits. *P* < 0.05, corrected for multiple comparisons, cluster size >3380 voxels

ANOVA analyses showed significant group differences in the vermis, tonsil and horizontal fissure of cerebellum, right middle frontal gyrus, left caudate lobe, left supplementary motor area, and right middle occipital gyrus (Fig. 1). The cluster size, F score, Z score, and coordinates of peak voxels for these clusters were listed in Table 2. Post hoc comparisons showed that the part of gray matter of the cerebellum, left putamen, hippocampus, parahippocampal gyrus, and bilateral caudate nucleus were all significantly reduced in the SZ-CD and the SZ-NCD patients, compared with that in the HCs, and that patients with cognitive deficits were found to have reduced gray matter density in the vermis and tonsil of cerebellum, left supplementary motor area, bilateral precentral gyrus compared with patients without cognitive deficits (Fig. 2). Decreased GMD in vermis and tonsil of bilateral cerebellum were found in SZ-CD group, while anterior and posterior lobe of left cerebellum in SZ-NCD group. The peak voxel details of these clusters were listed in Table 3.

**Table 2:** Regions showing gray matter density among SZ-NCD, SZ-CD, and control group

				MNI coordinates (mm, mm, mm)		
Cluster size	Brain regions	F value	Z value	X	Y	Z
8659	Cerebellum	14.79	4.28	−47	−59	−54
2259	Right MFG	14.37	4.22	33	20	41
8143	Left CL	13.36	4.07	−11	21	−2
	Left PHG	10.38	3.58	−21	−5	−27
3883	Left SMA	10.39	3.58	−6	−20	59
	Right PreCG	6.21	2.66	21	−21	66
6256	Right MOG	7.75	3.04	42	−68	5
	Right SOG	6.97	2.85	24	−72	27

X, Y, Z, coordinates of peak locations in the MNI space; F, Z, statistical value of peak voxel showing gray matter density differences among the three groups

*MFG* middle frontal gyrus, *CL* caudate lobe, *PHG* parahippocampal gyrus, *SMA* supplementary motor area, *PreCG* precentral gyrus, *MOG* middle occipital gyrus, *SOG* superior occipital gyrus. P < 0.05, corrected for multiple comparisons, cluster size >3554 voxels

**Table 3:** Significantly different clusters of gray matter density identified by post hoc analysis between SZ-CD, SZ-NCD, and HCs

					MNI coordinates (mm, mm, mm)		
Contrasts	Cluster size	Brain regions	T value	Z value	X	Y	Z
SZ-CD vs HCs	25,102	Cerebellum vermis, tonsil	4.43	4.04	−11	−57	−70
	3598	Right STG	3.29	3.11	68	−8	6
		Left PUT	2.97	2.84	−26	8	2
SZ-NCD vs HCs	8879	Left CL	5.02	4.49	−11	21	−2
		Left PHG	4.34	3.97	−21	−5	−27
	5924	Left Cerebellum	3.41	3.22	−42	−59	−48
SZ-CD vs SZ-NCD	8113	Left SMA	4.54	4.13	−6	−20	59
		Left PreCG	3.4	3.21	−26	−18	60
		Right PreCG	4.0	3.7	41	−15	36
	3383	Cerebellum vermis, tonsil	3.67	3.44	8	−44	−2

X, Y, Z, coordinates of peak locations in the MNI space

*STG* superior temporal gyrus, *PUT* putamen, *CL* caudate lobe, *PHG* parahippocampal gyrus, SMA, supplementary motor area, *PreCG* precentral gyrus, *MNI* Montreal Neurological Institute, *SZ-CD* schizophrenia with cognitive deficits, *SZ-NCD* schizophrenia with no cognitive deficits

P < 0.05, corrected for multiple comparisons, cluster size >3152 voxels (SZ-CD vs HCs), cluster size >3369 voxels (SZ-NCD vs HCs), cluster size > 3380 voxels (SZ-CD vs SZ-NCD)

**Fig. 3:**
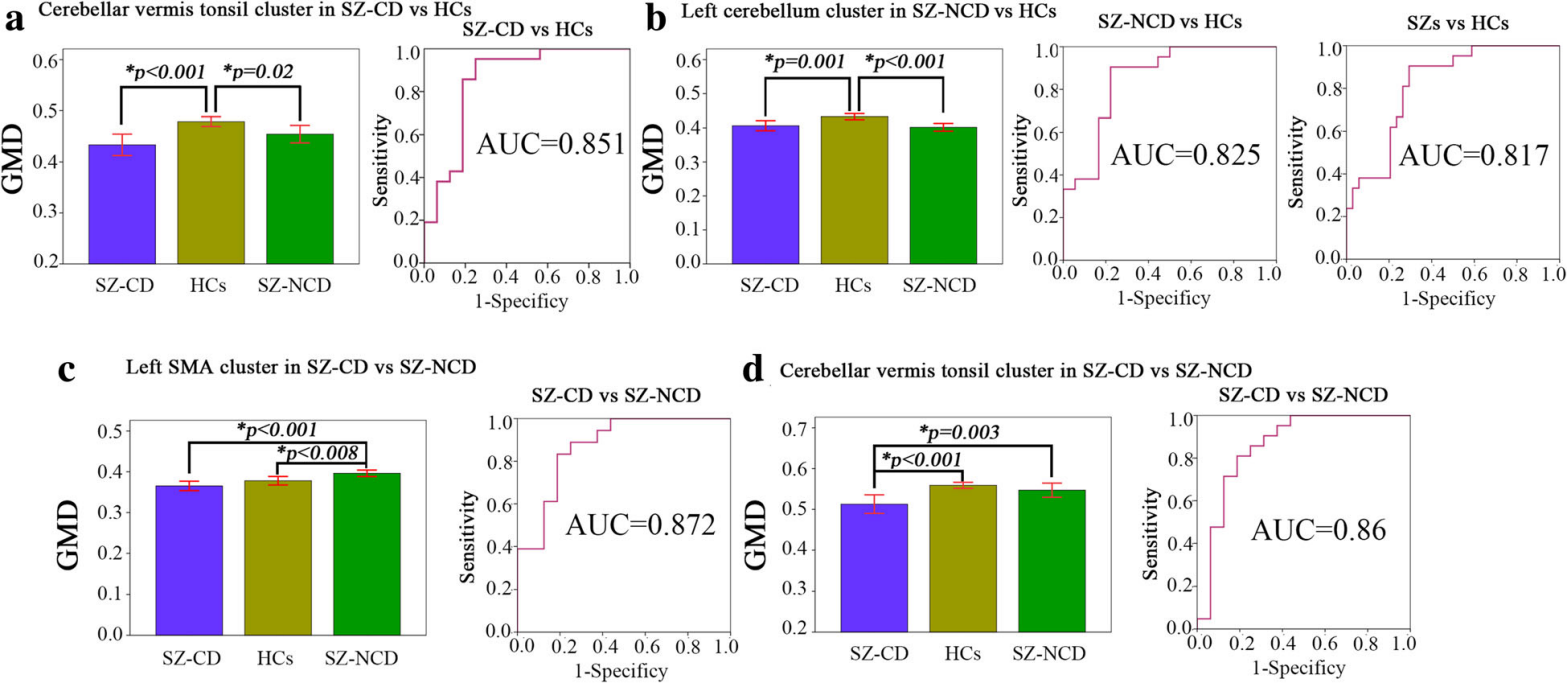
Significant differences of GMD in each cluster and receiver operating characteristic (ROC) curve for each pair of groups. Classifier results showed GMD of cerebellar vermis tonsil cluster in SZ-CD vs HCs could differentiate SZ-CD from controls (sensitivity,92.5%;specificity75%, **a**), the left cerebellum cluster (anterior lobe and posterior lobe) in SZ-NCD vs HCs could distinguish between SZ-NCD vs controls(sensitivity,90.5%;specificity,77.8%, **b**) and all SZ versus all controls (sensitivity,90.5%,specificity70.6%, **b**), left supplementary motor area (SMA)cluster(left SMA, bilateral precentral gyrus) in SZ-CD vs SZ-NCD could differentiate SZ-CD from SZ-NCD (sensitivity,83.3%;specificity,81.2%, **c**), GMD of cerebellar vermis tonsil cluster in SZ-CD vs SZ-NCD could differentiate SZ-CD from controls (sensitivity,92.5%;specificity75%, **d**) GMD, gray matter density; SZ-CD, schizophrenia with cognitive deficits; SZ-NCD, schizophrenia without cognitive deficits; HCs, healthy controls

For further quantitative analysis, we compared the mean GMD in significant clusters among the three groups and between each pair of groups. ANOVA results showed that the mean GMD of the cerebellar vermis cluster in SZ-CD vs HCs had a significant decrease in SZ-CD group compared with HCs group (*T* = 4.43, *P* < 0.001); left cerebellum cluster in SZ-NCD vs HCs, a significant decrease in SZ-NCD group (*T* = 3.41,P < 0.001); left SMA cluster in SZ-NCD vs SZ-CD, a significant decrease in SZ-CD group (*T* = 4.54,P < 0.001); cerebellar vermis cluster in SZ-NCD vs SZ-CD, a significant decrease in SZ-CD group (*T* = 3.67,*P* = 0.003). Furthermore, ROC showed GMD of cerebellar vermis tonsil cluster could differentiate SZ-CD from controls with a sensitivity of 92.5% and specificity of 75%, the left cerebellum cluster (anterior lobe and posterior lobe) could distinguish between SZ-NCD vs controls with a sensitivity of 90.5% and specificity of 77.8%, and all SZ versus all controls with a sensitivity of 90.5% and specificity of 70.6%,, left SMAcluster(left SMA, bilateral precentral gyrus) could differentiate SZ-CD from SZ-NCD with a sensitivity of 83.3% and specificity of 81.2% (Fig. 3).

### Correlation between the alterations in gray matter density and clinical symptom severity

**Fig. 4:**
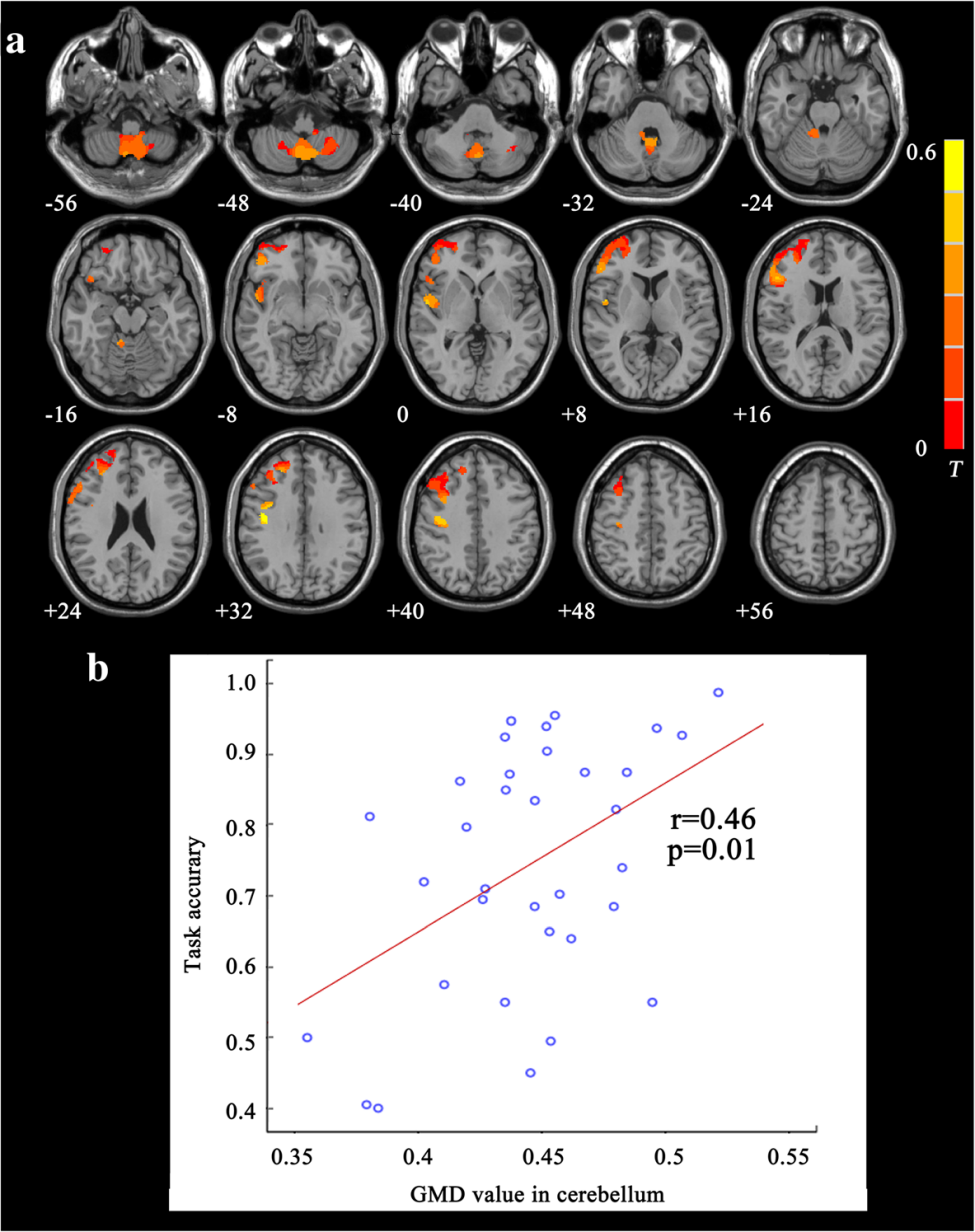
**a** Correlation maps of task accuracy and gray matter density for patients groups. Significant positive correlations were found in right precentral gyrus, posterior central gyrus, middle frontal gyrus, superior frontal gyrus, bilateral vermis and 8,9 of cerebellum. **b** The scatter plots between significant cluster in cerebellum and task accuracy (*r* = 0.46, *p* = 0.01), as assessed by 2 back task

In the combined patient groups, voxel–wise Pearson correlation maps showed positive correlations found in right superior frontal gyrus, vermis and tonsil of bilateral cerebellum, right precentral gyrus with task accuracy. GMD in bilateral cerebellum vermis cluster was positively correlated with task accuracy (*r* = 0.46, *p* = 0.014) (Fig. 4).

## Discussion

Based on VBM analysis, the present study found that first-episode SZ-CD had lower GMD in the vermis and tonsil of cerebellum than either SZ-NCD, or healthy controls and decreased GMD in left SMA cluster compared with SZ-NCD. Classification method revealed that the GMD of this cerebellum vermis cluster can be applied to differentiate SZ-CD patients from healthy controls and left SMA cluster differentiate SZ-CD group from SZ-NCD group with a high sensitivity and specificity. Further significant positive correlation was found between the GMD of this cerebellum vermis cluster and task accuracy.

In spite of the traditional viewpoint that the cerebellum only plays a role in motor-related function, recent studies have revealed that it contributes to cognitive processing and emotional control [13, 14]. The cerebellum is anatomically connected to frontal and parietal cortex by a cortico-cerebellar-thalamic-cortical circuit [15], and have been proven to have an important role in both motor and cognitive tasks through different connections depending on the demands of the task [13]. Cerebellar dysfunction, particularly in the vermis, has been proposed to lead to “cognitive dysmetria” in schizophrenia via the cortico-cerebellar-thalamic-cortical circuit [16].Reduced activation has been found in the cerebellar vermis during verbal working memory in schizophrenia [17].Although inconsistent findings of increased or decreased size or no change in vermis structure [18] in schizophrenia patients, volumetric alterations in the vermis have been associated with deficits in cognitive and executive function [19].Given the evidence that has begun to accumulate, the significantly abnormal GMD reported here in the vermis of SZ-CD patients compared with SZ-NCD patients and HCs is not surprising. A recent two-photon calcium imaging showed that cerebellar granule cells encode the expectation of reward, providing further important implications for cognitive processing in the cerebellum [20].Based on the fact that reduced GMD may be related to the loss of neurons, we speculate that cerebellar granule cells may be significantly disrupted in SZ-CD patients.

The relationship between the early changes of GMD in patient groups and clinical outcomes is of clinical importance. Reduced gray matter density of vermis was found to be positively related to the task accuracy in first-episode schizophrenia. The smaller the GMD the vermis, the more severely the patient’s cognitive ability is affected. As a classified variable, GMD of the vermis could differentiate the SZ-CD patients from healthy controls. The specific relationship between the reduced GMD in the vermis and cognitive impairment suggests that GMD changes in the vermis during the early stage of schizophrenia could sever as candidate biomarker for SZ-CD.

Our results also showed reduced GMD of superior temporal gyrus in SZ-CD group. Superior temporal gyrus was associated with auditory and speech comprehension. Decreased cortical thickness in superior temporal gyrus had been found in multiple sclerosis patients, which was correlated with cognitive performance [21]. In silent cerebral infarction patients, decreased gray matter volume was found and positively correlated with the MoCA scale [22]. In spite of different diseases, the relationships between structural abnormalities and cognitive impairment showed superior temporal gyrus was involved in cognitive function.

In addition, reduced GMD was also observed in the parahippocampal gyrus, left cerebellar posterior lobe in SZ-NCD patients group. The parahippocampal gyrus plays an important role in memory encoding and retrieval. A reduced GMD of the parahippocampal gyrus has also been found in other first-episode schizophrenia studies [23–26]. Another two morphological indexes, cortical thickness and gyrification have also be shown to be disturbed in the parahippocampal gyrus of first-episode schizophrenia [27] and chronically hallucinating schizophrenic patients [23]. Decreased GMD of left cerebellar posterior lobe was found in first-episode schizophrenia patients [28].Compared with previous studies, our study showed consistent results in first-episode schizophrenia patients.

Interestingly, much higher GMD value in left SMA cluster were found in SZ-NCD group than SZ-CD group and healthy controls, but no significant difference between SZ-CD and healthy controls. Meanwhile, left SMA cluster could distinguish SZ-CD group from SZ-NCD group. Researches had also reported that increased gray matter in SMA were found after aerobic exercise training [29], and which was associated with cognitive function [30].We speculated that increased GMD in SMA cluster might uncover structural compensation for cognitive function in SZ-NCD group.

Several limitations to our study should be considered. First, after dividing patients into subgroups based on their cognitive ability, there was a relatively small sample size of participants in each patient group. Further larger studies are required to replicate the current results. Second, the classification criterion was the mean accuracy in the N-back task. This task may not comprehensively represent one’s cognitive ability, although we added two other behavioral indicators to confirm our subgroup classification. Third, DSST and WAIS score of control group were did not estimated.

## Conclusions

The results of the present study provided new in vivo evidence for an early maturational deficit of the vermis in schizophrenia. We further found that reduced GMD in the vermis are correlated with cognitive deficits in first-episode schizophrenia. However, any inferences drawn from the present study require confirmation from further investigations in a larger sample size.

## Abbreviations

ANOVA: One-way analysis of variance
CPZ: Chlorpromazine
DSM-IV: Diagnostic and Statistical Manual for Mental Disorders Fourth Edition
DSST: Digital Symbol Substitution Test
FWHM: Full-width at half-maximum
GMD: Gray matter density
HCs: Healthy controls
SANS: Scale for the Assessment of Negative Symptoms
SAPS: Scale for Assessment of Positive Symptoms
SCID-I/P: Structured Clinical Interview for DSM-IV Axis I Disorders, Patient Edition
SD: Standard deviation
SPM8: Statistical Parametric Mapping 8
SZ-CD: Schizophrenia patients with cognitive deficits
SZ-NCD: Schizophrenia patients with no cognitive deficits
VBM: Voxel-based morphometry
WAIS_Digit: The Digit Symbol Substitution Test of the Wechsler Adult Intelligence Scale
WAIS-CR: Wechsler Adult Intelligence Scale Chinese Revised
WAIS-Information: The information subscale of the Wechsler Adult Intelligence Scale
